# Supplementary material for: Earth's earliest and deepest purported fossils may be iron-mineralized chemical gardens
Source: Proc Biol Sci. 2019 Nov 27;286(1916):20192410. doi: 10.1098/rspb.2019.2410 (PMC6939263; doi:10.1098/rspb.2019.2410)
Supplement: Figure S1 and Table S1 [file rspb20192410supp1.pdf]

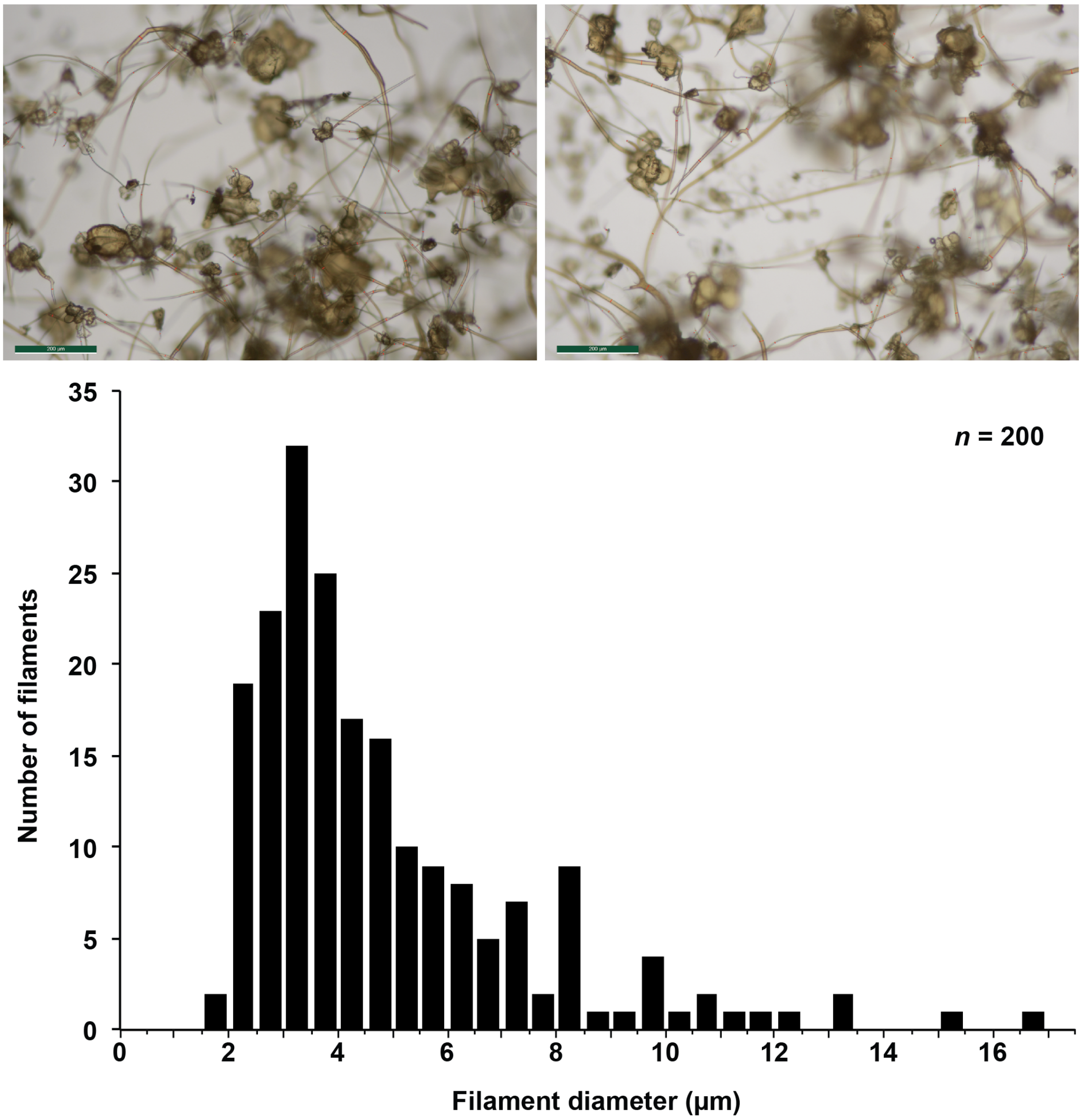

Figure S1: Chemical garden filaments grown from ferrous sulfate seed grains (sieved to <63 µm) dispersed in sodium silicate solution show a unimodal distribution of diameters with a positive skew. To produce the histogram, diameters of the 100 most in-focus filaments in each photomicrograph (top left and right; scalebars = 200 µm) were measured at their most in-focus point (marked in red) using ImageJ software calibrated to the Leica LAS software scalebars.

Table S1: Diameters measured on the photomicrographs shown in Figure S1

Left photomicrograph	Right photomicrograph
6.2	2.5
2.6	8.7
4.2	6.1
3	4.4
5	11.1
3.6	2.2
5.4	4
8.3	5.7
3	4.6
2.4	2.4
3.8	3.8
3.1	2.3
3.3	2.8
13.1	3.9
3.8	2.4
2.3	7.3
8	2.9
2.5	3.6
6.5	3.1
3.8	3.5
3.1	5.5
4.1	3.8
5.3	8
3.2	4.17
1.8	4.5
2.2	2.5
3.7	9.5
2.5	4.6
4.3	12.4
9.8	2.7
2.4	9.6
11.7	2.4
4.6	2.6
3	3.1
7.1	4.4
3.6	5
3.2	3.6
1.9	2.7
2.7	4.7
3.1	16.9
2.6	5.7
7.2	4.9
2.9	6.7

3.9	4.8
4.1	2.9
3.4	3.2
7.2	2.6
4.3	4
6.8	5.5
2.7	3.7
7.6	9.7
10.4	3.8
3.08	3.4
6	3.6
2.3	8.2
3.7	2.6
2.2	2.2
2.5	3.9
2.4	5.7
6.7	8.4
4.7	6
8.4	13
2.3	3.6
3.4	5.8
4.7	3
2.1	2.5
3.3	4.2
5.2	3
7.1	3.9
3.4	5.2
3.2	3.3
3.1	7.4
3	5.9
4.9	4.5
4.3	6.3
8	10.6
4.7	3.7
2.4	5.8
2	8.3
2.4	4.1
3.5	15.4
3.1	4.5
3	10.8
2.8	2.9
8.1	9
3.6	4.1
4.6	5.2
6.7	6
3	3

	4.4	7.2
	5.2	6.3
	2.6	4.6
	3.4	5.2
	7.8	3.7
	2	5.5
	2.6	6.3
	4.5	3.6
	4.1	3.2
	4.2	3.2
	3.2	5.4
